# Crosstalk between Gut and Brain in Alzheimer’s Disease: The Role of Gut Microbiota Modulation Strategies

**DOI:** 10.3390/nu13020690

**Published:** 2021-02-21

**Authors:** Umair Shabbir, Muhammad Sajid Arshad, Aysha Sameen, Deog-Hwan Oh

**Affiliations:** 1 Department of Food Science and Biotechnology, College of Agriculture and Life Sciences, Kangwon National University, Chuncheon 24341, Korea; umair336@gmail.com; 2 Department of Food Science, Faculty of Life Sciences, Government College University, Faisalabad 38000, Pakistan; msajidarshad@gcuf.edu.pk; 3 National Institute of Food Science and Technology, Faculty of Food, Nutrition and Home Sciences, University of Agriculture, Faisalabad 38000, Pakistan; ayshasameen@uaf.edu.pk

**Keywords:** gut dysbiosis, microbial metabolites, diet, probiotics, neurodegenerative diseases

## Abstract

The gut microbiota (GM) represents a diverse and dynamic population of microorganisms and about 100 trillion symbiotic microbial cells that dwell in the gastrointestinal tract. Studies suggest that the GM can influence the health of the host, and several factors can modify the GM composition, such as diet, drug intake, lifestyle, and geographical locations. Gut dysbiosis can affect brain immune homeostasis through the microbiota–gut–brain axis and can play a key role in the pathogenesis of neurodegenerative diseases, including dementia and Alzheimer’s disease (AD). The relationship between gut dysbiosis and AD is still elusive, but emerging evidence suggests that it can enhance the secretion of lipopolysaccharides and amyloids that may disturb intestinal permeability and the blood–brain barrier. In addition, it can promote the hallmarks of AD, such as oxidative stress, neuroinflammation, amyloid-beta formation, insulin resistance, and ultimately the causation of neural death. Poor dietary habits and aging, along with inflammatory responses due to dysbiosis, may contribute to the pathogenesis of AD. Thus, GM modulation through diet, probiotics, or fecal microbiota transplantation could represent potential therapeutics in AD. In this review, we discuss the role of GM dysbiosis in AD and potential therapeutic strategies to modulate GM in AD.

## 1. Introduction

The human body hosts trillions of microorganisms, including bacteria, fungi, archaea, and viruses. These symbiotic microorganisms can be beneficial, neutral or detrimental to the host and play regulatory functions in both health and disease. They can be found in the urogenital organs, respiratory tract, skin surface, and gastrointestinal tract (GIT). About 95% of the symbiotic microorganisms of the human microbiome reside in the gut [1]. Intricate ecological colonies of microorganisms dwell in the GIT and are collectively known as the gut microbiota (GM) [2,3]. The GM comprises mainly bacteria, fungi, bacteriophages, archaea, protozoa, and eukaryotic viruses and about 100 trillion microorganisms are harbored in the human GIT [4,5]. Firmicutes, *Bacteroides*, *Proteobacteria* and *Actinobacteria* represent the major bacteria of the gut [6], followed by *Bifidobacterium, Clostridium, Eubacterium, Peptococcus, Provetella*, etc. [7]. The small intestine consists of various types of bacteria, with content levels ranging from 10^4^ bacteria/mL to 10^6^–10^7^ bacteria/mL at the ileocecal junction, while the large intestine contains most of the non-spore forming bacteria (from 10^11^–10^12^ bacteria/g content) [8]. The population of GM is different for each person and is composed of various bacterial species. Furthermore, the composition of the GM is different at different stages of life. In older age individuals, increased Firmicutes and Bacteroidetes populations can be observed as compared to in younger individuals [9]. The GM is involved in metabolic processes and defense mechanisms and represents a dynamic and diverse population which impacts on the health and disease of the host. The GM develops the immune system in the intestinal mucosa and protects the host from carcinogens by releasing short-chain fatty acids (SCFA) [3]. Alterations of the GM community are referred to as dysbiosis [4,10], and these can lead to metabolic disorders. However, recent studies have proposed that this can also affect the central nervous system (CNS) because of the microbiota–gut–brain axis (MGBX) [11,12,13]. The brain regulates the secretory and sensory functions of the gut, and the connection between the gut and brain is interceded by physiological channels such as the autonomic nervous system, neuroendocrine system, neuroimmune pathways, and signaling molecules produced by the GM [14]. However, the actual mechanism and relationship between neural dysfunction and gut dysbiosis is elusive [15]. Emerging evidence suggests that gut dysbiosis can cause neurocognitive disorders such as schizophrenia, depression, bipolar disorder, anxiety, post-traumatic stress disorder, obsessive-compulsive disorder, and dementia, as well as the psychological and behavioral symptoms of dementia (Table 1) [12]. Additionally, metabolic syndromes and gut dysbiosis also contribute to Alzheimer’s disease (AD) and effect memory, learning, and hippocampal plasticity [16]. Diet, probiotics, and other therapeutic strategies have positive effects on GM modulation that may be helpful in the treatment of AD, as these factors alter the composition of the GM and have a positive impact on the host, improving the health status of the gut and body overall [3]. This review summarizes the role of GM dysbiosis, microbial metabolites, and metabolic impairment in AD. Additionally, the role of potential therapeutic strategies in modulating the GM composition and techniques to characterize the gut microbiome are also highlighted.

## 2. Impact of GM and Their Metabolites on the Brain

**Figure 1:**
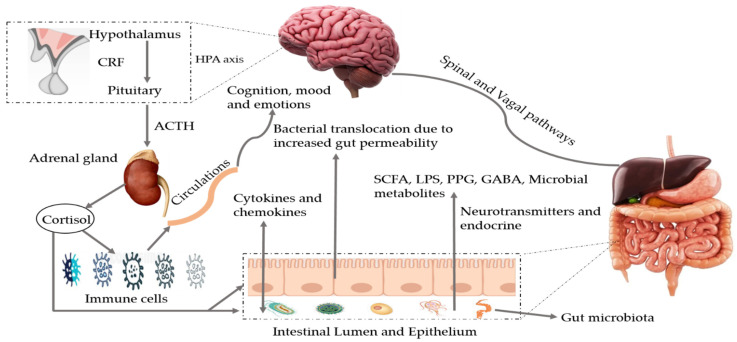
Bidirectional pathways involved in the communication between the gut microbiota and the brain (MGBX). They communicate through vagal and spinal nerves. SCFA, LPS, PPG, GABA, microbial metabolites, other neurotransmitters and endocrine cells are also involved. Dysbiosis can be caused by stress that may alter tryptophan levels, SCFA levels, the immune system, and gut permeability. Additionally, release of cytokines and chemokines (IL-6, IL-1β, IL-8) can lead to neuroinflammation and activation of HPA axis. SCFA: short-chain fatty acids, LPS: lipopolysaccharides, PPG: peptidoglycans, GABA: gama-aminobutyric acid, HPA axis: hypothalamic–pituitary–adrenal axis, CRF: corticotropin-releasing factor, ACTH: adrenocorticotropic hormone.

During metabolic processes, the GM can produce several bioactive metabolites that can enter into the bloodstream via absorption into enterohepatic circulation [4]. Metabolites linked to the phenotype of a disease can be recognized by nuclear magnetic resonance (NMR) and mass spectrometry-based metabolomics of body fluids such as urine, feces, or plasma. This makes it possible to carry out joint analyses of the host phenotypes, metabolome, and microbiome to identify mechanistic links [31]. The GM metabolizes a plethora of neurotransmitters and neuromodulators (such as short-chain fatty acids (SCFA), gamma-aminobutyric acid, acetylcholine, dopamine, glutamate, and serotonin) [32,33,34]. Microbial species such as *Saccharomyces*, *Bacillus*, *Lactobacillus*, *Escherichia*, and *Bifidobacterium* are known to produce these types of neurotransmitters [33]. Preliminary human studies have revealed that bacterial-based interventions can also change neurotransmitter levels involved in synaptic plasticity (including brain-derived neurotrophic factor), and regulate the activity of *N*-methyl-d-aspartate and serotonin receptors [34]. Impairment of the GM composition or their metabolites modulates the gut–brain axis [33] and regulates cognition, memory, mood, and social behavior [35,36]. Moreover, dysbiosis may result in the formation of toxic misfolded proteins with a β-sheet conformation that promotes loss of synaptic connections, cellular cell dysfunction, and neurodegeneration [37]. The pathways involved in MGBX are illustrated in Figure 1. Moreover, some of the major microbial metabolites (neurotransmitters) and their role in brain health are exhibited in Table 2.

## 3. Alzheimer’s Disease

Dementia is a group of symptoms or a syndrome that causes deterioration in memory, behavior, thinking, ability to perform daily activities, judgement, and language. Dementia generally affects the elderly people but is not classified as a normal part of aging [54]. Among different kinds of dementia, AD is the most common [55], and it contributes to 60–80% of dementia cases [56]. It is one of the rapidly growing brain diseases [13] and it has been reported that AD and other types of dementia represent the 5th most prominent reason for deaths around the globe. Around 50 million people suffer from dementia, and this is expected to double by 2030, and triple by 2050. Every year, approximately 10 million additional cases are reported worldwide [54,57]. In the early stages of AD, people may suffer from memory lapses such as forgetting familiar locations and words, while the middle-stage is the longest stage that can last for years and the person may become angry or frustrated, confused, and act unpredictably. In the last stage, individuals lose the ability to carry on conversations, respond to their environment, and ultimately lose control of movement. As cognitive and memory conditions continue to be exacerbated, individuals require extensive care due to significant personality changes [54,56]. Various immune-inflammatory variations have been found in patients with AD and mild cognitive impairment (MCI), including raised levels of pro-inflammatory cytokines and activated microglia that allow crosstalk between the peripheral and central immune systems [58]. The major hallmarks of AD are as follows: development of amyloid beta (Aβ) plaques (Aβ peptides and Aβ-oligomers) and neurofibrillary tangles in the nerve cells [59]; elevated generation of reactive oxygen species leads to neuroinflammation and cell death. Additionally, vascular abnormalities and mitochondrial damage also contribute to the pathogenesis of AD [60,61].

## 4. Metabolic Impairment and AD

The GM may contribute to metabolic health, but dysbiosis in GM composition triggers metabolic syndrome. Metabolic syndrome is a combination of abnormalities contributing to different diseases such as malnutrition, non-alcoholic liver disease, obesity, cardio-metabolic diseases and type-2 diabetes [4,62,63]. Deficiency of SIRT3, known as a mitochondrial deacetylase, is a significant cause of metabolic syndrome. SIRT3 regulates the functioning of critical mitochondrial proteins by deacetylation [64,65]. Tyagi et al. [66] stated that deficiency of SIRT3 increases the formation of amyloid plaques and induces neuroinflammation in the brain. They crossed SIRT3^−/−^ mice with APP/PSI mice (double-transgenic mouse models of AD expressing a chimeric mouse/human amyloid precursor protein and containing the L166P mutation, both directed to the CNS [67]) and generated APP/PS1/SIRT3^−/−^ mice with metabolic syndrome and amyloid pathology. Aggravation of glucose intolerance, insulin resistance, deposits of Aβ, and hallmarks of neuroinflammation such as tumor necrosis factor (TNF)-α, interleukin (IL)-1β, and cyclooxygenase-2 were observed in the generated mice. Additionally, activated and proliferated microglial cells were also reported. Thus, hypothetically, metabolic syndrome and the induced amyloid pathology may interact with age related disorders such as diabetes, cardiovascular diseases, obesity, and hypertension and coexist with AD. Additionally, Gupta et al. [68] revealed that type-2 diabetes mellitus contributes to the pathophysiology of AD. Thomas et al. [69] agreed with this and stated that studied diabetic subjects (*n* = 69) suffered from at least 1 AD risk factor (e.g., cognitive decline, Aβ deposition, hyperphosphorylated tau, and genetic susceptibility). On the other hand, obesity is also associated with morphological and functional impairment in mitochondria that initiate insulin resistance (responsible for tau hyperphosphorylation and Aβ aggregation) peripheral inflammation, memory deficits, and oxidative stress that increase the risk of AD [70,71,72]. Cuomo et al. [73] disclosed that PCR and NMR analyses showed that *Helicobacter pylori* could potentially modulate AD, type-2 diabetes, cardio-metabolic disease, and obesity. *Helicobacter pylori* induces high levels of amino acids and activates the mammalian target of rapamycin complex 1 (regulates the host’s metabolism) and branched-chain amino acids. Furthermore, *Helicobacter pylori* toxin VacA resides within the mitochondria, contributing to the depletion of ATP, oxidative stress, and causes fragmentation of these organelles that induces autophagy and endure bacterial colonization of gastric mucosa [74]. It also modulates the inflammation of hyperphosphorylation of tau proteins and stimulates Aβ formation.

## 5. GM Dysbiosis and AD

**Figure 2:**
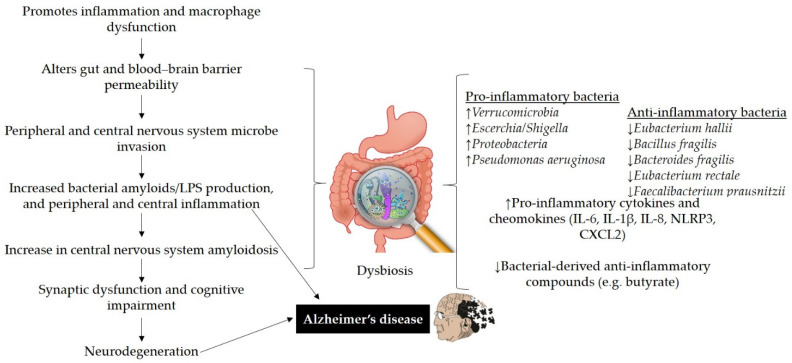
The possible role of gut dysbiosis in neurodegeneration and Alzheimer’s disease. Disturbance in gut homeostasis impairs gut permeability due to the action of pro-inflammatory bacteria that produce bacterial amyloids/LPS and cause macrophage dysfunction. Amyloids and LPS can increase inflammatory cytokines (IL-6, IL-1β, IL-8, NLRP3, CXCL2). Impairment in gut and blood–brain barrier may lead to the deposition of Aβ fibrils in the brain and can contribute to AD pathogenesis. LPS: lipopolysaccharides, IL: interleukin, NLRP3: nod-like receptor protein 3, CXCL2: C-X-C motif chemokine ligand 2.

The GM has various links to inflammatory and metabolic pathways. Dysbiosis affects the synthesis of signaling proteins that influence metabolic processes related to AD progression [15]. Aging alters the GM composition (high abundance of pro-inflammatory bacteria than anti-inflammatory bacteria) and induces local systematic inflammation that causes impairment in the permeability of the GIT and blood–brain barrier function [3]. *Peptostreptococcaceae*, *Clostridiaceae*, *Bifidobacteriaceae*, *Turicibacteraceae*, *Mogibacteriaceae*, and *Ruminococcaceae* families were found to be less abundant as compared to *Bacteroidaceae*, *Gemellaceae*, and *Rikenellaceae* families in AD participants [75]. More specifically, it has been stated that dysbiosis contributes to the enhancement of pro-inflammatory bacteria (such as *Verrucomicrobia*, *Escerchia/Shigella*, *Proteobacteria*, and *Pseudomonas aeruginosa*) and decreases the abundance of anti-inflammatory bacteria (such as *Eubacterium hallii, Bacillus fragilis, Bacteroides fragilis, Eubacterium rectale*, *Faecalibacterium prausnitzii*, and *Bifidobacterium)*, potentially promoting neuroinflammation and worsening the formation of Aβ plaques [76]. Decreases in microbial diversity have been reported in a microbiome study of AD and MCI human patients, with progressive growth of *Enterobacteriaceae*, *Enterobacteriales*, and *Gammaproteobacteria* being observed in comparison to controls. Moreover, enhanced biosynthesis and glycan metabolism, decreases in immune system-related pathways, and decreases the abundance of *Ruminococcaceae, Lachnospiraceae*, *Firmicutes*, and *Clostridiaceae* were also noted in patients [77]. On the other hand, Lee et al. [78] revealed that a transgenic murine model of AD showed significant changes in phyla (e.g., Bacteroidetes and Firmicutes), while an increase in *Clostridium leptum* group was also observed [79]. Additionally, dysbiosis may promote AD symptoms, such as oxidative stress and insulin resistance [11]. Hypothetically, it has been stated that the GM might modulate the oxidative state of the CNS through the produced metabolites. Suppressed levels of butyrate could enhance mitochondrial dysfunction resulting in the production of reactive oxygen species [80]. Cerovic et al. [81] stated that gut dysbiosis leads to both central and peripheral pathological events that could possibly increase the risk of AD. Dysbiosis in 5xFAD mice was associated with the progression of the CCAAT/enhancer binding protein β/asparagine endopeptidase pathway that mediated AD pathology through cleaving both Aβ precursor and Tau proteins [82]. In a recent study, Li et al. [83] used RNA sequencing, Y maze, transcriptome sequencing, Gene Expression Omnibus, and quantitative reverse-transcriptase PCR techniques for APP^swe^/PS1^ΔE9^ transgenic mice and wild-type mice to examine the role of dysbiosis in AD. They found a significantly different composition of GM, decreased cognitive ability, and increased amyloid formation. Microbiota-mediated intestinal and systemic immune aberrations trigger the pathogenesis of AD in ADLP^APT^ mice [84]. Thus, it can be concluded that impairment of the GM is correlated with decreases in cognitive function and might play role in the enhancement of the amyloid deposition via stimulating the mitogen-activated protein kinase signaling pathways (these control a wide range of cellular processes, such as apoptosis, differentiation, proliferation, and stress responses [85]) in the brain. Taken together, the possible role of gut dysbiosis in contributing to neurodegeneration and AD is illustrated in Figure 2.

### Bacterial Amyloids and Lipopolysaccharides in AD

Amyloids are unique proteins with self-aggregation properties, and their accumulation can cause cellular dysfunction [86]. Initiation of Aβ in the brain is elusive, however, different in vitro and in vivo studies have claimed that amyloids produced by GM may cross-seed Aβ deposition [87]. Bacterial strains, such as *Escherichia coli*, *Bacillus subtilis*, *Salmonella Typhimurium*, *Pseudomonas fluorescens*, *Staphylococcus aureus*, etc., are considered to produce amyloids. These strains produce curli, TasA, CsgA, FapC, phenol soluble modulins, etc., amyloids that promote the misfolding of Aβ fibrils and oligomers. The production of amyloid proteins helps bacterial cells to bind to each other and form biofilms to resist destruction by immune or physical factors [88]. It has been documented that bacterial amyloids are different from the amyloids in the brain in terms of their primary structure, but share similarities in their tertiary structure [89]. Exposure to bacterial amyloid proteins in the gut may cause priming of the immune system that, in turn, increases the immune responses to the endogenous production of neural amyloids in the CNS [88,90]. Bacterial amyloids, through molecular mimicry, may act as prion proteins that evoke cross-seeding in which the amyloidogenic protein causes the production of another protein, such as a host protein with a different primary structure, to adopt the pathogenic β-sheet structure [88]. Osorio et al. [91] proposed that amyloids are antigens that generate a defensive response to Aβ deposition in order to suppress danger signals. CsgA shares dissimilarity in sequences with Aβ_42_ but is similar in triggering AD-related pathogenic effects and promoting cerebral plaque deposition [81]. In a recent study, Javed et al. [92] claimed that FapCS favorably bound with Aβ, showed a catalytic capacity in seeding peptide amyloidosis, impaired cognitive performance, and behavior pathology in vitro, in silico and in a zebrafish AD model. Additionally, phenol soluble modulins contain cross-α structure and form cross-β fibrils associated with AD [93]. Sampson et al. [86] exhibited that curli-producing *Escherichia coli*, enhance the pathology of amyloid α-synuclein (involved in the progression of AD, dementia and Parkinson’s disease [94]) in the gut and brain of mice. In a similar study, increased expression of Toll-like receptor 2, and pro-inflammatory mediators IL-6 and TNF, in association with astrogliosis and microgliosis, were also observed in rats exposed to the curli amyloid. Cattaneo et al. [76] stated that amyloidosis positive patients exhibited higher serum levels of IL-1β, IL-6, C-X-C motif chemokine ligand, and nod-like receptor protein 3, and lower serum levels of anti-inflammatory cytokine IL-10.

Lipopolysaccharides (LPS) mainly produced by Gram-negative bacteria (Proteobacteria and Bacteroidetes: pro-inflammatory bacteria) can induce inflammation and disrupt the blood–brain barrier function [95]. A plethora of in vivo and in vitro studies suggested that LPS activate several intracellular molecules that change the expression of different inflammatory mediators and in turn, contribute or initiate the progression/development of neurodegeneration [96]. Khan et al. [97] stated that LPS activate the Toll-like receptor 4 and cause the epithelial and intestinal-wall inflammation that results in leaky gut. LPS also activate astrocyte and microglial cells in the GIT that secrete pro-inflammatory cytokines, those later gain entry to the bloodstream through leaky gut. This serum LPS cause disruption in the blood–brain barrier and may enter the brain and reactivate microglia, and astrocytes, and various amyloid genic and inflammatory pathways. Increased inflammatory cytokines and nuclear factor kappa B (NF-κB) leads to an increase in amyloid precursor protein and Aβ protein cleavage and accumulation that causes the death of neurons and AD development. Moreover, LPS promote Aβ_42_ fibrillogenesis which triggers the formation of Aβ_1–40/42_ plaques in the white and grey matter of AD brains [98]. The abundance of LPS was also observed in the hippocampus and in the neocortex [99], and in the lysates from the superior temporal lobe of AD brains [100]. A study suggested that LPS treatment in mice contributed to neuroinflammation, cognitive impairment and sickness behavior with neuronal loss in the hippocampus and activated microglia. The levels of IL-1β, TNF-α, nitric oxide, and prostaglandin E2 were increased with the activation of the NF-κB signaling pathway [101]. Thingore et al. [102] reported that LPS administration enhanced neuroinflammation and contributed to oxidative stress through a decrease in reduced glutathione, superoxide dismutase, and increases in lipid peroxidation in the brains of mice. Treatment with 2,4,6-trinitrobenzenesulfonic acid in mice caused colitis and increased membrane permeability, levels of LPS, Enterobacteriaceae (including *Escherichia coli*) with a decrease in *Lactobacillus johnsonii* in the GM composition, activated NF-κB and TNF-α, and displayed cognitive impairment. On the other hand, treatment with *Lactobacillus johnsonii* restored the GM dysbiosis, levels of LPS in the blood, memory and cognitive impairment [103]. Thus, bacterial LPS and amyloids contribute to the hallmarks of AD through MGBX and restoration of GM homeostasis could be beneficial for treating AD.

## 6. Potential Therapeutic Strategies for AD

### 6.1. Diet and Food Components

Different epidemiological studies suggest a strong correlation among lifestyle-related factors, diet, and the onset and consolidation of AD and other kinds of dementia [13]. Studies have reported that nutritional interventions might be helpful to mitigate or delay the risk of cognitive impairment, AD and other non-psychiatric comorbidities. Fieldhouse et al. [104] stated that a suboptimal diet is related to severely impaired cognition, attributed to low vegetable consumption and is pronounced in AD and dementia. Intake of a diet rich in probiotics, plant-based foods, polyphenols, vitamins, antioxidants, and ω-3 polyunsaturated fatty acids can delay AD [105], as diet can shape host-associated GM composition [106]. Additionally, low intake of saturated fats, refined sugars, and animal-derived proteins is also considered beneficial in this regard [105]. Moreover, healthy dietary patterns can be beneficial for cognitive health and can show neuroprotective properties. The Mediterranean diet is deemed beneficial for AD patients as it is rich in many components such as legumes, fruits, vegetables, cereals, polyphenols, non-digestible carbohydrates, and unsaturated fatty acids [107]. Polyphenols (PPs) are naturally occurring bioactive compounds and the most abundant forms of antioxidants in the human diet [63]. PPs have been recognized as potent agents that can lower the risk of AD [13]. Furthermore, vitamins have a positive effect on AD due to their role in preventing oxidative stress and inflammation that would otherwise lead to Aβ and tau phosphorylation [108,109]. The gut microbiome can produce vitamins that are necessary for brain health (including vitamin B6, B9, B12, etc.) [97]. Park et al. [110] exhibited that deficiencies of folate and vitamin B12 are associated with impaired memory function and hippocampal insulin signaling in AD rats. Thus, supplementation of vitamins (such as vitamin B complex) may also be helpful in delineating a treatment of AD. Prebiotics promote the growth of beneficial bacteria (including *lactobacillus* and *bifidobacteria*), and improve dysbiosis and the associated inflammatory conditions that may ameliorate cognitive impairment [111]. Conversely, the Western diet, consisting of saturated and high trans-fat, high sugar with fewer vegetables and fruits is considered a nutrient-imbalanced diet pattern [107]. This diet may cause metabolic syndromes and diseases and can increase the chances of AD development. A recent study by Gabriel et al. [112] stated that chronic exposure of a high-fat, high-sugar Western diet to C57BL/6N mice significantly contributed to obesity and memory impairment. These dietary patterns increase the abundance of *Flavobacterium, Runella,* and *Flectobacillus* that can activate inflammatory responses by regulating IL-1β and the NF-κB pathways [113]. Ye et al. [114] reported that diets with high-fat can affect the gut–brain axis in zebrafish and that this could be due to modulation of the GM. For example, the abundance of *Acinetobacter* is <0.1% of the total GM in normal conditions and it is associated with inflammatory responses in the gut. However, diets with high-fat content can enhance its abundance by 100-fold [115], which may lead to LPS production and neuroinflammation.

On the other hand, melatonin (a hormone produced by the pineal gland) can prevent or slow down the progression of AD. Melatonin has the potential to enhance Aβ clearance through glymphatic-lymphatic drainage, blood–brain barrier transportation and degradation pathways, and ameliorates Aβ-induced neurotoxicity [116]. It has been stated that melatonin controls the GM, and its treatment can increase the ratio of *Firmicutes* to *Bacteroidetes* and *Akkermania* [117] and decrease the pathogenic bacteria in gut [118]. Melatonin levels were observed to be lower in AD subjects as compared to healthy aged subjects. Thus, supplementation of melatonin can exert neuroprotective effects on the brain. Additionally, administration of melatonin can reduce Aβ plaques and enhance cognitive performance [116]. The role of diet and other food components in AD is demonstrated in Table 3.

### 6.2. Probiotics

Probiotics are microorganisms present in the human gut and when supplied in adequate quantities confer health benefits to the host [129]. Diet-based interventions include administration of probiotics through specific supplements, supplementation with PPs, probiotic enriched foods, consumption of dietary fiber, and foods rich in prebiotics [130]. Probiotics can secrete and produce bacterio-toxins (such as bacteriocins) which can suppress bacterial invasion and block pathogen adhesion to epithelial cells [131]. Probiotics compete with pathogenic bacteria for nutrients and binding sites [132]. Additionally, they increase the integrity of the barrier and mucus production by exerting a trophic effect on intestinal mucosa and effects on epithelial cell cytokine secretion [33,133]. Oral bacteriotherapy with probiotics has been recently identified to treat and prevent many pathologies [134,135]. A systematic review suggested that preliminary animal studies support the potential role of probiotics in improving cognitive functioning, possibly by decreasing the levels of oxidative and inflammatory biomarkers in MCI or AD; *Lactobacillus* and *Bifidobacterium* strains could be the most promising candidates (Table 4). Additionally, the effectiveness of probiotics administration could be affected by dosage, the proportion of each strain, severity of patients and so forth [129].

### 6.3. Fecal Microbiota Transplantation

Fecal microbiota transplantation (FMT) is being studied as a potential intervention for many conditions [136]. FMT is the process of transferring prescreened stool into the GIT of patients to restore the function and increase the overall diversity of GM [137]. Fecal material is expected to be from a highly organized stool bank and administrated through colonoscopy, enema, or capsule [138]. At present, FMT is widely accepted for the treatment of *Clostridium difficile* infections, however, trials related to human diseases such as cancer, neurodegenerative diseases, and inflammatory bowel disease to metabolic diseases are still ongoing worldwide. FMT could be beneficial for cognitive impairment and decreasing Aβ plaques in AD animals’ brains [139]. However, studies suggest that caution should be taken in the premature extrapolation of data from preclinical studies because of the inherent limitations of rodent models. Standardization, stool bank services and management, safety assessment, etc., have still not been significantly upgraded, causing complex challenges for regulators and clinicians. Stool availability, adverse effects, and poorly or undefined mechanisms of action are the major concerns related to FMT [11]. Additionally, Sood et al. [140] documented that FMT safety with other routes of administration, patient acceptance, and tolerability must be assessed before recommending this method as the most appropriate way to administer FMT (Table 4).

## 7. Techniques to Characterize the GM

Several techniques are currently being used to understand the role of GM in both inducing and preventing specific pathological conditions. The 16S rRNA sequencing method represents the mainstay in terms of a regular and standardized approach to evaluating the composition of human microbiome taxa species down to the genus level [152]. However, next-generation sequencing technologies such as Ion Torrent, Illumina sequencing, and Roche 454 pyrosequencing can identify species composition using shorter DNA stretches with a higher sequence coverage [153]. Computational approaches to identify the 16S rRNA sequencing of disease or non-disease causing microbes have also been developed to better evaluate the biology of the GM community [154]. On the other hand, using 16S rRNA sequencing makes it challenging to understand the genomic information of species with low abundance. Thus, recent studies have been shifting towards the use of high-throughput data techniques for obtaining quantitative and qualitative information of metabolites, DNA, mRNA transcripts, and proteins of the microbial groups in the microbiome [152]. Shotgun metagenomic sequencing helps analyze the whole genomic DNA of bacteria to understand and identify the functional potential of microbial genes [155]. Additionally, meta-omic approaches can be helpful in providing a comprehensive functional view of microorganisms and their role within the microbiome [156]. The HMP Unified Metabolic Analysis Network is another example that can perform functional and metabolic reconstructions of metagenomic data [157]. Although high-throughput next-generation sequencing methods are advantageous, they are also expensive and complex.

## 8. Limitations and Future Perspectives

Most of the animal studies cite here, explored the possible interaction between GM composition and cognitive function. However, human studies were performed on subjects with an established diagnosis of dementia, and outside the geriatric constructs of MCI and cognitive frailty. Thus, a substantial gap exists between the interventional and observational studies performed in animal models of AD. Additionally most of the clinical studies have been conducted with small samples, which is why comprehensive profiling of GM composition and functionality is lacking. Longitudinal studies of high-risk populations are required to examine temporal changes in the GM, as direction of causality cannot be based via cross-sectional studies. Implementation of neuroimaging variables and blood biomarkers as secondary outcomes can enable us to better understand the GM and cognitive functioning.

GM metabolites may affect the host cellular pathways involved in differentiation, maturation, and proliferation functions [33]. Well-defined methodological standards are needed to compare the studies, and other variables such as drugs, concomitant pathologies, and diet must be carefully considered in analyses [158]. Many studies related to uncovering the role of the GM in AD are limited by the fact that only the 16S rRNA sequencing method is being used, providing data on taxa down to the genus level. Because of this, important associations at the strain or species level may be lost. Identifying and characterizing the role of specific microbiomes should be a primary focus of MGBX research. The use of high-throughput next generation sequencing methods for metagenomics, metabolomics, and informatics will be helpful in managing the databases deriving from ongoing GM studies. However, successful results depend on the treatment, disease stage, proper combination of nutrients and bacterial strains [130]. Diet, probiotics and FMT are considered to represent potential therapeutics for AD, MCI, and other neurodegenerative diseases. However, there are still limitations, especially in probiotic and FMT interventions. Cox et al. [159] stated that caloric restriction experiments exhibited that *Lactobacillus* and *Faecalibaculum* genera protected mice from the development of Aβ plaques, while the Lachnospiraceae family and *Bacteroides* genus were associated with higher Aβ levels in the brain. However, these findings are only apparent in female mice, not male mice; thus, further studies are needed to explore this issue. Other factors such as sex may significantly impact GM links to neurodegeneration. Halverson et al. [12] concluded that not all studies have shown effective therapeutic responses because studies have used different treatment methods, study conditions, times of treatments, dosages, and probiotic strains. Therefore, research is needed to better understand the underlying mechanisms, specific procedures, and guidelines to enhance the effectiveness of GM modulation.

## 9. Conclusions

Evolving evidence from animal and human studies suggests that dysbiosis affects the GIT and its related diseases and can also contribute to neurodegeneration due to the fact that the gut communicates with the brain through the gut–brain axis. The GM can produce neurotransmitters that can influence the relations hip between the neurochemistry of the brain and brain disorders such as mood, cognition, behavior, etc. Interestingly, the fungal taxa of the GM with gut bacteria also correlates with AD markers [120]. Diet, probiotics, and FMT administration can modulate the GM composition, alleviating the pathological changes associated with AD. From the safety point of view, diet is one of the most suitable interventions to modulate dysbiosis, but more research is required in this area before application in clinical practice. Thus, further studies are required to understand the specific effects of these therapeutics in the prevention or alleviation of AD.

## Figures and Tables

**Table 1:** Changes in microbiome occur during several mental conditions and their related findings.

Condition	Study	Change in Microbiome	Findings	Reference
Depression	Human (*n* = 90)	↑Phylum Bacteroidetes, classes Gammaproteobacteria and Bacteroidia, order Bacteroidales, and genera *Flavonifractor* and *Sellimonas.* ↓Phylum Firmicutes, class Clostridia, order Clostridiales, family Ruminococcaceae (*Subdoligranulum, Faecalibacterium*, *Ruminococcus 1,* and *(Eubacterium) coprostanoligenes*), and family Christensenellaceae (*Christensenellaceae R7 group*).	Low levels of SCFA, anti-inflammatory, and butyrate-producing bacteria may link the GM and the low-grade and chronic inflammation.	[17]
	Human (*n* = 16)	Depression showed a positive correlation with *Paraprevotella*, negative correlations with *Clostridiales*, *Clostridia*, *Firmicutes*, and the RF32 order.	Intestinal inflammation and integrity markers found to be related to the response to treatment in patients with symptom severity and major depressive disorder.	[18]
	Human (*n* = 111)	Clostridiale*s* order, Ruminococcaceae family (*Clostridium symbiosum* and *Coprococcus catus*) variably differentiated depression severity strata.	*Coprococcus catus* specifically found to be a contributor to psychiatric functioning.	[19]
Schizophrenia	Human (*n* = 26)	↑Proteobacteria, *Chaetomium* ↓*Faecalibacterium*, Lachnospiraceae, and *Trichoderma.*	*Faecalibacterium* and Genera Lachnospiraceae allow opportunistic pathogens such as Protobacteria to translocate and are involved in CD4 cell differentiation and can increase gut TH17 cells that can penetrate through the BBB and induce abnormal behavior.	[20]
	Animal	↑*Lactobacillus* and *Bifidobacterium.* ↓*Akkermansia*	Administration of Inulin modulated GM decreased 5-HT and inflammatory cytokines and enhanced BDNF though the MGBX and ameliorated schizophrenia.	[21]
Bipolar disorder	Human (*n* = 36)	↓*Bifidobacteria* to *Enterobacteriaceae* ratio ↑*Enterobacter spp*, *Faecalibacterium prausnitzii*, *Clostridium Cluster IV, Atopobium Cluster*, and *Bacteroides-Prevotella group.*	Brain-gut coefficient of balance (B-G_CB_) was used as a new concept.	[22]
	Human (*n* = 32)	↑Actinobacteria Coriobacteriia ↓*Faecalibacterium* and *Ruminococcaceae.*	*Actinobacteria* and *Coriobacteriia* take part in lipid metabolism correlating with cholesterol levels, found in bipolar patients.	[23]
Anxiety	Human (*n* = 9)	↓Microbial richness and diversity. ↑*Escherichia-Shigella, Fusobacterium,* and *Ruminococcus gnavus.*	Enhanced gut permeability due to decrease in SCFA producing bacteria. Increase in the abundance of *Escherichia-Shigella, Ruminococcus gnavus,* and *Fusobacterium* bacteria may support systemic inflammation and degrade mucins.	[24]
Post-traumatic stress disorder	Animal	Alteration in Bacteroidetes, Firmicutes, Proteobacteria, and Cyanobacteria levels.	Changes in levels of neurotransmitters such as 5-HT, dopamine, and norepinephrine were observed in stressed rats.	[25]
	Human (*n* = 93)	↑*Escherichia/Shigella* and *Enterococcus* ↓Autochthonous taxa, Lachnospiraceaeae and Ruminococcaceae.	*Escherichia/Shigella* and *Enterococcus* linked with poor cognition, and higher levels of lipopolysaccharides are linked with neuroinflammation through MGBX.	[26]
Obsessive-compulsive disorder	Human (*n* = 43)	↓species richness/evenness (Inverse Simpson, α-diversity), relative abundance of butyrate producing genera (*Anaerostipes, Odoribacter,* and *Oscillospira*).	Mean C-reactive protein, but not IL-6 and TNF-α, was increased in the patients. C-reactive protein exhibited mild to strong linkage with psychiatric symptomatology.	[27]
Dementia	Human (*n* = 128)	↑Enterotype I and III bacteria were associated with dementia.	Serum triglycerides, serum C-reactive protein, and markers of insulin resistance were found in subjects. Fecal lactic acid and ammonia were linked to dementia.	[28,29]
	Human (*n* = 77)	↓*Clostridia* and its phylum Firmicutes and the *Ruminococcus, Ruminococcaceae*, *Clostridiales* at order, family and genus levels.	Decrease in SCFA producing bacteria and indole-3-pyruvic acid was recognized as a signature for prediction and discrimination of AD.	[30]

↑: Higher, ↓: Lower, AD: Alzheimer’s disease, BDNF: brain-derived neurotrophic factor, GM: gut microbiota, BBB: blood–brain barrier, MGBX: microbiota–gut–brain axis, 5-HT: 5-hydroxytryptamine, SCFA: short-chain fatty acids, IL: interleukin, TNF-α: tumor necrosis factor-α. B-G_CB_: the ratio of (oxygenated hemoglobin)/(Bifidobacteria to Enterobacteriaceae ratio) to analyze the relationship between brain function and GM. n: number of total subjects in the study but the columns of GM and findings are only showing the data of diseased ones.

**Table 2:** Neurotransmitters produced by gut microbiota and their role in brain function.

Gut Microbiota	Metabolites	Study	Association in Brain Function	References
*Lactobacillus*, *Bifidobacterium*	GABA	Human/animal metagenomic	Main inhibitory neurotransmitter of the CNS and a potential mediator between bacterial cells and the host. Regulates depression, anxiety, behavioral and cognitive functions.	[38,39]
*Akkermansia muciniphila, Lactobacillus plantarum DR7*	Serotonin or 5-HT	Animal metagenomic	Regulates mood, learning, cognition, and memory.	[40,41]
*Bifidobacterium longum*, *Clostridium symbiosum*, *Faecalibacterium prausnitzii*, *Lactobacillus fermentum*	SCFA	Human/animal metagenomic	Regulate neuro-immunoendocrine function, reduce inflammation, promote the synthesis and secretion of neurotransmitters, hormones and suppress permeability of the blood–brain barrier.	[42,43,44]
*Lactobacillus, Escherichia, Streptococcus, Lactococcus, Bacillus*	Dopamine	Human/animal metagenomic	Protects neuron loss, improves motor deficits, cognition, reduced stress and anxiety.	[45,46,47]
*Escherichia coli, Morganella morganii, Lactobacillus vaginalis, Enterobacter aerogenes*	Histamine	Human/animal metagenomic	Regulates depression-like behaviors and impaired sleep-wake cycle.	[48,49,50]
*Coryneform, Bacteroides vulgatus, Campylobacter jejuni, Lactobacillus*	Glutamate	Human/animal metagenomic	Play role in molecular mechanism of learning, memory, and synaptic plasticity.	[51]
*Lactobacillus, Bacillus*	Acetylcholine	Animal metagenomic	Memory, emotional personality, self-care ability, cognition, and social life ability.	[52,53]

GABA: gamma-aminobutyric acid, CNS: central nervous system, SCFA: short-chain fatty acids, 5-HT: 5-hydroxytryptamine.

**Table 3:** Role of diet and other food components in AD related to gut microbiome.

Intervention	Study	Findings	Reference
MMKD	Human (*n* = 17)	MMKD ↑Abundance of *Erysipelotriaceae*, *Slackia*, *Enterobacteriaceae*, *Christensenellaceae,* and *Akkermansia* while ↓ abundance of *Bifidobacterium* and *Lachnobacterium* in subjects. Fecal butyrate and propionate negatively correlated with Aβ_42_, while *Proteobacteria* positively correlated with Aβ_42_:Aβ_40_ in patients with MCI and AD. MMKD slightly decreased fecal acetate and lactate and increased butyrate and propionate.	[119]
MMKD	Human (*n* = 17)	↑proportion of families *Togniniaceae, Phaffomyceteceae, Cystofilobasidiaceae, Sclerotiniaceae, Trichocomaceae,* and genera *Kazachstania, Botrytis, Cladosporium,* and *Phaeoacremonium* while ↓*Meyerozyma* in patients with MCI. Fungal taxa showed distinct correlation arrays with AD markers. MMKD ↑*Mrakia* and *Agaricus* while ↓*Claviceps* and *Saccharomyces*.	[120]
Curcumin	APP/PS1 double transgenic mice (*n* = 15)	Curcumin administration altered the abundance of *Rikenellaceae, Bacteroidaceae, Lactobacillaceae*, and *Prevotellaceae* at family level, and *Parabacteroides, Bacteroides*, and *Prevotella* at genus level (many of them are considered to be associated with AD development). Moreover, curcumin intervention decreased Aβ plaque formation, enhanced memory abilities and spatial learning. Metabolites of curcumin are also reported to exhibit neuroprotective ability.	[121]
Q3G	C57BL/6J mice (*n* = 30)	Q3G administration restored Aβ_1–42_-induced cognitive impairment, GM dysbiosis and reduced SCFA production. Ameliorated Tau phosphorylation and Aβ accumulation, restored cAMP response element-binding protein and BDNF levels in the hippocampus.	[122]
EGCG	C57BL/6 wild-type and APP/PS1 mice (*n* = 30)	Treatment with EGCG improves the peripheral parameters like liver insulin pathway signaling, and central memory deficits. It increased cAMP response element binding phosphorylation rates and synaptic markers. Additionally, EGCG significantly reduced Aβ formation and plaque by enhancing the levels of α-secretase in brain and reduced neuroinflammation.	[123]
Palmitoylethanolamide and luteolin	Sprague Dawley rat	Prevented the Aβ-induced microgliosis and astrogliosis, and upregulated the gene expression of pro-inflammatory cytokines and enzymes. Additionally, it prevented the reduction in GDNF and BDNF mRNA levels.	[124]
ω-3 Fatty Acid	Human (*n* = 174)	Higher ω-3 fatty acid plasma levels were related to the lower rate of cognitive deterioration.	[125]
Xylooligosaccharides	APP/PS1 mice	Effectively decreased GM alteration and cognitive dysfunction attenuated inflammatory responses and ameliorated the tight junction barrier in the hippocampus and intestine.	[126]
FOS	APP/PS1 mice	Ameliorated pathological changes and cognitive deficits, upregulated the expression levels of synapsin I and postsynaptic density protein 95, and decreased the phosphorylated level of c-Jun N-terminal kinase. Additionally, FOS administration reversed the altered GM density.	[127]
Inulin	*ApoE3* (*E3*FAD) and *ApoE4* (*E4*FAD) mice (*n* = 17)	↑Beneficial GM, acetate, propionate, and butyrate in blood and cecum, energy production and blood metabolites in citric acid cycle and pentose phosphate pathway that support nucleic acid and nucleotide biosynthesis; ↓the pro-inflammatory rate in the context of reduced α-diversity in E4FAD-inulin mice.	[128]

↑: Higher, ↓: Lower, AD: Alzheimer’s disease, MCI: mild cognitive impairment, MMKD: Modified Mediterranean ketogenic diet, Q3G: Quercetin-3-*O*-Glucuronide, GM: gut microbiota. SCFA: short-chain fatty acids, EGCG: Epigallocatechin-3-gallate, BDNF: brain-derived neurotrophic factor, GDNF: Glial cell-derived neurotrophic factor, FOS: Fructooligosaccharides. *n*: number of total subjects in the study but the column of findings is only showing the data of diseased ones.

**Table 4:** Use of probiotics and FMT to modify the GM in AD and related findings.

Intervention	Study	Findings	Reference
*Lactobacillus plantarum C29*-fermented soybean (DW2009)	Human (*n* = 100)	Administration of DW2009 enhanced the serum BDNF levels that may significantly improve cognitive and memory functions.	[141]
VSL#3	APP^NL-G-F^ mice	Increased in the serum SCFA (lactate, acetate, butyrate, propionate, and isobutyrate). Both serum and brain levels of acetate and lactate in mice correlated with increased expression of the neuronal activity marker.	[142]
SLAB51	3xTg-AD mice	Oral administration ameliorated glucose uptake by restoring the brain expression levels of GLUT1 and GLUT3, and IGF receptor β, in accordance with reduced phosphorylation of AMPK and Akt. Additionally, decreased phosphorylated tau aggregates and increased glycated hemoglobin and the accumulation of advanced glycation end products in mice and improved memory.	[143]
*Bifidobacterium longum* (NK46)	5xFAD-transgenic mice	Oral administration ameliorated GM composition, decreased blood and fecal LPS levels, increased tight junction protein expression in the colon and suppressed TNF-α expression and NF-κB activation. Additionally, decreased the cognitive decline, β/γ-secretases, Aβ accumulation, and caspase-3 expression in the hippocampus of mice.	[144]
*Clostridium butyricum*	APP/PS1 transgenic mice	Treatment restored the GM impairment and butyrate. It prevented Aβ accumulation, cognitive impairment, production of TNF-α, IL-1β, and microglia activation.	[145]
*Lactobacillus lactis* strain carrying one plasmid (pExu)	3xTg-AD mice	Oral administration ameliorated memory, decreased levels of Aβ peptides, modulated ubiquitin-proteasome system and autophagy, and reduced neuronal inflammatory and oxidative processes.	[135]
*Bifidobacterium breve* A1 (MCC1274)	Human (*n* = 79)	Probiotic treatment improved the visuospatial/constructional, immediate memory, and delayed memory.	[146]
5 *Lactobacillus* and 5 *Enterococcus strains*	C57BL/6J male mice	Reduction in leaky gut by increasing tight junctions, and decreasing inflammation. This study concluded that probiotics could prevent or treat aging-related leaky gut and inflammation that leads to AD.	[147]
*Lactobacillus plantarum* ATCC 8014	APP/PS1 mice	Decreased Αβ levels in the hippocampus, ameliorated cognitive deterioration and protected neuronal integrity and plasticity	[148]
*Bacteroides thetaiotaomicron*	APP/PS1TG mice	Decreased Αβ levels in the hippocampus and significantly improved memory function.	[149]
*Multispecies probiotics*	Human (*n* = 23)	Decreased the inflammation-causing bacteria and fecal zonulin concentrations, and enhanced serum kynurenine concentrations.	[150]
FMT	ADLP^APT^ transgenic mice	Mice showed reductions in chronic intestinal and systemic inflammation and loss of epithelial barrier integrity. FMT ameliorated the formation of glial reactivity, neurofibrillary tangles, Aβ plaques, and cognitive impairment. Additionally, abnormalities in intestinal macrophage activity were also reversed.	[84]
FMT	APPswe/PSEN1dE9 transgenic mice	Increase in synaptic plasticity, synapsin I expression, decrease in Aβ_40–42_, tau protein phosphorylation, COX-2 and CD11b levels were observed after FMT. It also restored GM impairment and short-chain fatty acids levels.	[139]

SCFA: short-chain fatty acids, BDNF: brain-derived neurotrophic factor, AMPK: adenosine monophosphate-activated protein kinase, GLUT: glucose transporter, IGF: insulin-like growth factor, Akt: protein-kinase B, GM: gut microbiota, TNF-α: tumor necrosis factor alpha, LPS: lipopolysaccharides, NF-κB: nuclear factor kappa B, IL: interleukin-1β, FMT: fecal microbiota transplantation, COX-2: cyclooxygenase-2, CD11b: cluster of differentiation molecule 11B, *n*:number of total subjects in the study but the column of findings is only showing the data of diseased ones. VSL#3: a probiotic formulation (commercially available) having 8 bacterial strains: 4 strains of *Lactobacillus* (*L. delbrueckii* subspecies *bulgaricus, L. casei*, *Lactobacillus plantarum*, and *L. acidophilus*), 3 strains of *Bifidobacterium* (*B. infantis, B. breve,* and *B. longum*), and strain of *Streptococcus* (*S. salivarius* subspecies *thermophilus*) [151]. SLAB51: a probiotic mixture having 8 bacterial strains *L. brevis* DSM 27961, *L. acidophilus* DSM 32241, *L. helveticus* DSM 32242, *L. paracasei* DSM 32243, *L. plantarum* DSM 32244, *B. lactis* DSM 32246, *B. lactis* DSM 32247, *S. thermophilus* DSM 32,245 [143]. Multispecies probiotics: two types of capsules each containing 3 bacteria either *L. fermentum, L. plantarum*, and *B. lactis,* or *L. acidophilus, B. bifidum, and B. longum.*

## Data Availability

No new data were created or analyzed in this study. Data sharing is not applicable to this article.

## Abbreviations

5-HT	5-hydroxytryptamine
ACTH	Adrenocorticotropic hormone
AD	Alzheimer’s disease
Akt	Protein-kinase B
AMPK	Adenosine monophosphate-activated protein kinase
Aβ	Amyloid beta
BBB	Blood–brain barrier
BDNF	Brain-derived neurotrophic factor
CD11b	Cluster of differentiation molecule 11B
CNS	Central nervous system
COX-2	Cyclooxygenase-2
CRF	Corticotropin-releasing factor
CXCL2	C-X-C motif chemokine ligand 2
EGCG	Epigallocatechin-3-gallate
FMT	Fecal microbiota transplantation
FOS	Fructooligosaccharides
GABA	gama-aminobutyric acid
GDNF	Glial cell-derived neurotrophic factor
GIT	Gastrointestinal tract
GLUT	Glucose transporter
GM	Gut microbiota
HPA axis	Hypothalamic–pituitary–adrenal axis
IGF	Insulin-like growth factor
IL	Interleukin
LPS	Lipopolysaccharides
MCI	Mild cognitive impairment
MGBX	Microbiota–gut–brain axis
MMKD	Modified Mediterranean ketogenic diet
NF-κB	Nuclear factor kappa B
NLRP3	Nod-like receptor protein 3
PPG	Peptidoglycans
PPs	Polyphenols
Q3G	Quercetin-3-*o*-glucuronide
SCFA	Short-chain fatty acids
TNF-α	Tumor necrosis factor-α
